# Single-Nucleotide Polymorphisms (SNP) Mining and Their Effect on the Tridimensional Protein Structure Prediction in a Set of Immunity-Related Expressed Sequence Tags (EST) in Atlantic Salmon (*Salmo salar*)

**DOI:** 10.3389/fgene.2019.01406

**Published:** 2020-02-27

**Authors:** Eva Vallejos-Vidal, Sebastián Reyes-Cerpa, Jaime Andrés Rivas-Pardo, Kevin Maisey, José M. Yáñez, Hector Valenzuela, Pablo A. Cea, Victor Castro-Fernandez, Lluis Tort, Ana M. Sandino, Mónica Imarai, Felipe E. Reyes-López

**Affiliations:** ^1^ Department of Cell Biology, Physiology and Immunology, Faculty of Biosciences, Universitat Autònoma de Barcelona, Barcelona, Spain; ^2^ Centro de Genómica y Bioinformática, Facultad de Ciencias, Universidad Mayor, Santiago, Chile; ^3^ Escuela de Biotecnología, Facultad de Ciencias, Universidad Mayor, Santiago, Chile; ^4^ Centro de Biotecnología Acuícola, Departamento de Biología, Facultad de Química y Biología, Universidad de Santiago de Chile, Santiago, Chile; ^5^ Facultad de Ciencias Veterinarias y Pecuarias, Universidad de Chile, Santiago, Chile; ^6^ Facultad de Ciencias, Universidad de Chile, Santiago, Chile

**Keywords:** single-nucleotide polymorphism, immune response, synonymous SNP, nonsynonymous SNP, homology modeling, 3D protein structure, molecular dynamics simulation, *Salmo salar*

## Abstract

Single-nucleotide polymorphisms (SNPs) are single genetic code variations considered one of the most common forms of nucleotide modifications. Such SNPs can be located in genes associated to immune response and, therefore, they may have direct implications over the phenotype of susceptibility to infections affecting the productive sector. In this study, a set of immune-related genes (*cc motif chemokine 19 precursor* [*ccl19*], integrin β2 (itβ2, also named *cd18*), *glutathione transferase omega-1* [*gsto-1*], *heat shock 70 KDa protein* [*hsp70*], *major histocompatibility complex class I* [*mhc-I*]) were analyzed to identify SNPs by data mining. These genes were chosen based on their previously reported expression on infectious pancreatic necrosis virus (IPNV)-infected Atlantic salmon phenotype. The available EST sequences for these genes were obtained from the Unigene database. Twenty-eight SNPs were found in the genes evaluated and identified most of them as transition base changes. The effect of the SNPs located on the 5’-untranslated region (UTR) or 3’-UTR upon transcription factor binding sites and alternative splicing regulatory motifs was assessed and ranked with a low-medium predicted FASTSNP score risk. Synonymous SNPs were found on *itβ2* (c.2275G > A), *gsto-1* (c.558G > A), and *hsp70* (c.1950C > T) with low FASTSNP predicted score risk. The difference in the relative synonymous codon usage (RSCU) value between the variant codons and the wild-type codon (ΔRSCU) showed one negative (*hsp70* c.1950C > T) and two positive ΔRSCU values (*itβ2* c.2275G > A; *gsto-1* c.558G > A), suggesting that these synonymous SNPs (sSNPs) may be associated to differences in the local rate of elongation. Nonsynonymous SNPs (nsSNPs) in the *gsto-1* translatable gene region were ranked, using SIFT and POLYPHEN web-tools, with the second highest (c.205A > G; c484T > C) and the highest (c.499T > C; c.769A > C) predicted score risk possible. Using homology modeling to predict the effect of these nonsynonymous SNPs, the most relevant nucleotide changes for *gsto-1* were observed for the nsSNPs c.205A > G, c484T > C, and c.769A > C. Molecular dynamics was assessed to analyze if these GSTO-1 variants have significant differences in their conformational dynamics, suggesting these SNPs could have allosteric effects modulating its catalysis. Altogether, these results suggest that candidate SNPs identified may play a crucial potential role in the immune response of Atlantic salmon.

## Introduction

Genetic variation occurs within and among populations, leading to polymorphisms that could be associated with genetic trait or also a phenotype in the presence of an environmental stimulus (Brookes, 1999; Rebbeck et al., 2004; Hirschhorn and Daly, 2005). A single-nucleotide polymorphism (SNP) is a single genetic code variation (i.e., polymorphic). Although multiallelic SNPs do exist, the SNPs are usually biallelic (two alternative bases occur) and require a minimum frequency (>1%) in the population (Wang et al., 1998). The SNPs are the most common form of variation in the genome and they are extensively used to study genetic differences between individuals and populations. These SNPs may contribute to changes in the genomic sequence, either in the coding (exons), intergenic, or noncoding (introns) region (Dijk et al., 2014; Ahmad et al., 2018).

SNPs are considered the most useful biomarkers for disease diagnosis or prognosis due to their common frequency, ease of analysis, low genotyping costs, and the possibility to carry out association studies based on statistical and bioinformatics tools (Srinivasan et al., 2016). Thus, SNPs have gained importance as major drivers in disease-association studies in the recent era. In mammals, on the past decade it has been seen an enormous progress in identifying hundreds of thousands SNPs to identify associations with complex clinical conditions and phenotypic traits associated with hundreds of common diseases (Welter et al., 2014; Wijmenga and Zhernakova, 2018).

Furthermore, SNPs may also have a great influence on the immune response towards pathogenic challenges and diseases outcome, contributing in a range of susceptibility to infections among the individuals. Thus, the SNP may have a protective role, may influence the rate of diseases progression or even the type of cellular immune response evoked by pathogens (Hill, 2001; Skevaki et al., 2015). In this regard, polymorphisms on several immune-related genes have been associated with susceptibility to infections including *pattern recognition receptors* (*prr*) and downstream signaling molecules (Skevaki et al., 2015), *mannose- binding lectin 2* (*mbl2*) and *toll-interleukin 1 receptor domain containing adaptor protein* (*tirap*) (Gowin et al., 2018), *c-c chemokine receptor type 5* (*ccr5*) (Martin et al., 1998; Salkowitz et al., 2003; Fellay et al., 2009; Chapman and Hill, 2012), interleukin 6 (*il-6*) (Zhang et al., 2012), and *il-22* (Zhang et al., 2011), among others.

In species related to aquaculture, SNPs are especially important because they may be associated with different phenotypic traits with economical implications. Therefore, this increase of information has a direct impact on the accuracy of selection for these traits, improving the rate of genetic gain and production efficiency (Hayes et al., 2007b). The ubiquity of SNPs across the genomes examined to date, has allowed their use as markers for a wide range of applications including quantitative trait locus (QTL) mapping, pedigree analysis, association studies and population genetics, among others. Conversely, whereas the effect on gene mutations in mammals has been well documented, such information in teleost species is still limited. However, several efforts have been made to provide information regarding the consequences of genetic alterations in immune response-related genes and may influence susceptibility to diseases in fish (Kongchum et al., 2011). In this context, SNP variations were found on *il-1β* of *Cyprinus pellegrini* and *C. carpio* that can be helpful in understanding differential resistance to koi herpesvirus (KHV) and *Aeromonas hydrophila,* respectively (Jia et al., 2015; Wenne, 2018). On the other hand, three SNPs were identified in the *leukocyte cell-derived chemotaxin-2* (*lect2*) gene to be associated with resistance to the big belly disease on *Latis calcarifer* (Fu et al., 2014a; Wenne, 2018). Three SNPs in the *mast cell protease 8* (*mcp-8*) gene were also significantly associated with resistance of tilapia to *Streptococcus agalactiae* (Fu et al., 2014b; Wenne, 2018).

In Atlantic salmon, some studies have reported SNPs associated with a resistance genotype against infectious pancreatic necrosis (IPN) virus (IPNV). IPN is a highly transmissible disease with worldwide distribution that occurs both at the initial stage of rearing in freshwater and in post-smolts in seawater (Bruno, 2004). Importantly, asymptomatic carriers (McAllister et al., 1993), establishment of viral persistence (Reyes-Cerpa et al., 2014), IPN-resistance phenotype (Reyes-López et al., 2015), and vertical transmission through eggs (Bootland et al., 1991) have been reported. In this matter, several segregating SNP markers linked to a major QTL associated with resistance against infectious pancreatic necrosis virus (IPNV) in Atlantic salmon from an Scotland commercial breeding program have been reported (Houston et al., 2012; Wenne, 2018). Similarly, a QTL in Norwegian salmon has been employed in marker-assisted selection in breeding companies from Norway and Scotland, which resulted in 75% reduction in the number of IPN-outbreaks in the salmon farming industry. This QTL has been located on the SNP-based linkage map and identified as the epithelial *cadherin* (*cdh1-1*) gene with a functional involvement in viral attachment and entry of IPNV (Moen et al., 2009; Moen et al., 2015; Wenne, 2018). Based on these reports, it seems that the strategy to detect SNPs in immune-related genes could provide a set of candidate polymorphisms that could explain the correlation between the pathogen and the disease phenotype. Particularly, the relevance to identify SNPs in the coding sequence of immunity-related genes could explain directly (causal) the variability upon a specific phenotype evaluated (Carlson et al., 2003; Cheng et al., 2004; Hirschhorn and Daly, 2005). In this context, most of the knowledge about fish immune response is based on large-scale expressed sequence tag (EST) sequencing that has helped to identify immune-related genes in teleosts. Undoubtedly, the EST sequencing based on tissues that play a central role on immune response contribute to detect gene sequences that are directly related to host defense functions. The identification of a set of splenic leukocytes immune-related genes from Atlantic salmon IPNV-infected using EST analysis has been reported (Cepeda et al., 2011; Cepeda et al., 2012). Importantly, some of these genes were detected differentially expressed when the IPN-susceptible and IPN-resistant phenotypes were compared (Reyes-López et al., 2015). Thus, the search and identification of SNPs on these immune-related genes using a data mining strategy may contribute to provide a set of candidate polymorphisms that could help in the progress to establish a link between the possible causes of this differential expression pattern and the IPN-phenotype variability. Hayes et al. (Hayes et al., 2007b), described for first time an *in silico* detection of 2,507 putative SNPs in Atlantic salmon from the alignment of 100,866 EST. Despite the large number of SNPs identified, there is no gene directly associated with immune function.

Therefore, the aim of this study was the identification of SNPs (*in silico*) by data mining upon a set of immune-related genes whose expression has been previously reported in response to the infection with IPNV in Atlantic salmon. For this purpose, a set of immune-related genes (*cc motif chemokine 19 precursor* [*ccl19*], *integrin β2* (*itβ2*, also named *cd18*), *glutathione transferase omega-1* [*gsto-1*], *heat shock 70 KDa protein* [*hsp70*], *major histocompatibility complex class I* [*mhc-I*]) were selected as target for the *in silico* SNP search and identification using as template the EST sequences for these genes obtained from the Unigene database. Based on their nucleotide sequence, the SNPs were located in the 5’/3’-UTR or in the translated region. In the case of those nucleotide variations located in the translated region, the SNP were classified as synonymous (sSNPs) or nonsynonymous (nsSNPs) based on the change provoked in the predicted amino acid sequence. While for sSNPs a codon usage analysis was conducted, for those nsSNPs a homology modeling analysis was carried out in order to evaluate whether they could have an effect on the predicted tridimensional protein structure. In addition, on those nsSNP in which a significant change in the three-dimensional protein structure was observed by homology modeling, an analysis of such variants over the time was performed by molecular dynamics (MD) simulation. This study provide a set of identified candidate SNPs that may help to determine potential correlations between the immunity gene expression pattern in Atlantic salmon and their response against the pathogens they are exposed under aquaculture conditions.

## Material and Methods

### Selection of Genes Modulated in Response to IPNV and Sequence Cluster Collection

The immune-related genes analyzed (*cc motif chemokine 19* precursor [*ccl19*], *integrin β2* [*itβ2*], *glutathione transferase omega-1* [*gsto-1*], *heat shock 70 KDa protein* [*hsp70*], *major histocompatibility complex class I* [*mhc-I*]) were selected based on their previously reported expression on splenic leukocytes isolated from IPNV-infected Atlantic salmon and whose expression was also differentially modulated between the IPN-susceptible and IPN-resistant phenotypes (Reyes-López et al., 2015). The EST sequences for the previously above-mentioned selected candidate genes were downloaded from the Unigene database (NCBI). The detail regarding all the EST sequences analyzed in this study to identify SNPs are indicated on **Supplementary Tables 1**–**5**.

### Data Mining for SNP Identification

The identification of SNPs by data mining was carried out in order to identify possible functional effects in the sequences related to defense and immune response in Atlantic salmon challenged with IPNV. All the sequences including in the analysis were first preanalyzed in order to remove any vector sequences or repetitive elements using Cross-Match (Ewing and Green, 1998) and RepeatMasker (Tarailo-Graovac and Chen, 2009), respectively. The search for SNPs was carried out based on multiple sequence alignment including the total number of sequences collected for each evaluated gene using the HaploSNPer web-based tool (Tang et al., 2008). From the alignment analysis, each nucleotide variant detected was considered as putative SNP and it was defined according to the nucleotide position in the gene sequence. The sequences were then filtered to exclude possible sequencing errors and noninformative polymorphisms (variants with a frequency lower than 1%). The nonfiltered SNPs were chosen as the most probable or reliable SNPs. The nucleotide variations were described according to the nomenclature suggested by Dunnen and Antonarakis (Den Dunnen and Antonarakis, 2001).

### Effect of SNPs on Gene Function

In order to determine the SNP location onto the gene region (5’-UTR, coding region, 3’-UTR), the nucleotide sequence was obtained based on their Unigene annotation from Nucleotide database (NCBI) and compared them by alignment with BioEdit sequence alignment editor (version 7.0.5.3). In addition, the nucleotide sequence was also used as template to get the predicted amino acid sequence using ORF Finder tool (NCBI) to confirm by protein BLAST (NCBI) the annotation for the unigene sequence analyzed.

The functional impact of the SNP was assessed depending on the gene region (5’-UTR, coding region, 3’-UTR) on which the nucleotide variant was located. For those SNP located in the noncoding region (5’-UTR, 3’-UTR) the predictive nucleotide variant effect upon motifs associated to transcription factor binding sites was evaluated with TFSearch webtool (http://diyhpl.us/~bryan/irc/protocol-online/protocol-cache/TFSEARCH.html) (Heinemeyer et al., 1998). Furthermore, the predictive SNP effect onto possible exon splicing enhancer [ESEfinder (http://krainer01.cshl.edu/cgi-bin/tools/ESE3/esefinder.cgi?process=home) (Cartegni et al., 2003); RESCUE-ESE (http://hollywood.mit.edu/burgelab/rescue-ese/) (Fairbrother et al., 2004)] and exon splicing silencer [FASS-ESS (http://genes.mit.edu/fas-ess/) (Wang et al., 2004)] was also evaluated. Based on these results, the SNP functional effect was predicted according to FASTSNP in order to assign a FASTSNP score (Yuan et al., 2006). A FASTSNP score between 0 and 5 was assigned to each individually SNP evaluated (representing from 0 to 5 the minimum to maximum functional SNP effect, respectively). On the other hand, for those SNP located in the coding region the nucleotide variant was first individually evaluated with ORF Finder tool (NCBI) in order to compare the predicted unigene amino acid sequence with the SNP-containing unigene sequence by BioEdit sequence alignment editor (version 7.0.5.3). In the case of the nucleotide variations located in untranslated region (5’-UTR; 3’-UTR) and those did not provoke any change in the predicted amino acid sequence (sSNP; synonymous amino acid change), the SNP effect was evaluated with the FASTSNP decision tree in order to assign a FASTSNP score (Yuan et al., 2006). In addition, on each sSNP identified a codon usage analysis was performed according to Sharp et al. (M.Sharp and Li, 1987). The difference in the relative synonymous codon usage (RSCU) value between the variant codons and the wild-type codon (ΔRSCU = RSCU mutant - RSCU wild-type) was calculated for all the SNPs identified based on the codon usage database (http://www.kazusa.or.jp/codon) for *Salmo salar*. On the other hand, in those nucleotide variants in which a change in the amino acid predicted sequence was detected (nsSNP; nonsynonymous amino acid change), the nucleotide variation impact was analyzed combining the score obtained from Sorting Intolerant From Tolerant (SIFT) (applied to human variant databases) (http://sift.bii.a-star.edu.sg/) (Ng and Henikoff, 2003) and POLYPHEN web-based resources (Flanagan et al., 2010). The results were ranked according to the protocol established by Bhatti et al. (Bhatti et al., 2006) with some modifications in order to homogenize the significance of all scores obtained for the SNP obtained in this study (lower score = minimum effect; higher score = maximum effect). Thus, the scores obtained for SIFT were ranked from I (tolerated) to IV (intolerant); meanwhile the scores obtained for POLYPHEN were ranked from A (benign) to E (probably damaging). The combination of the SIFT and POLYPHEN analysis give a score whose value was ranked from 1 (minimum effect) to 4 (maximum effect). The implications of the nonsynonymous SNPs reported at protein tertiary structure level was also evaluated *in silico* through homology modeling analysis.

### Homology Modeling

In order to evaluate whether the nonsynonymous SNPs reported in this study have an effect on the predicted tridimensional protein structure, a homology modeling analysis was carried out. For this, the nucleotide sequence was used as target to obtain the predicting tridimensional protein structure with the CPHModels-3.0 webserver (Nielsen et al., 2010). The higher bit score alignment obtained was chosen as template for GSTO-1 (PDB ID: 1EEM; score = 278; E-value = 4e-75). Alternatively, the predicted protein structure for CCL19 (PDB ID: 2HCI; score = 55; E-value = 2e-08), ITB2 (PDB ID: 2KCN; score = 42; E-value = 2e-04), HSP70 (PDB ID: 1YUW; score = 1036; E-value = 0) and MHC class I (PDB ID: 1KTL; score = 182; E-value = 4e-46) were also obtained. The tridimensional protein structure for each PDB ID match was obtained to then be used as template for the homology modeling of the above mentioned gene sequences using the MODELLER webtool (Swiss-Model) (Sali and Blundell, 1993). The stereochemical quality of the modeled tridimensional protein structure was evaluated with Procheck webtool (Swiss-Model) (Laskowski et al., 1996).

### All-Atom Explicit Solvent MD Analysis

The protonation state of residues at physiological conditions (pH = 7) was assigned using the *Propka* software included in the Maestro Suite (Olsson et al., 2011). The models were solvated in TIP3P truncated octahedron with an extension of 12 Å over the protein surface, including 4 Cl^-^ atoms to maintain the net charge neutrality within the system. Parameters for calculations were derived from the AMBER *ff14SB* forcefield (Maier et al., 2015). Energy minimization was carried out in four different stages, in each one of them, 5,000 steps of steepest descent followed by 5,000 steps of a conjugated gradient were performed. In the first stage, the solute was fixed with a positional restraint of 500 kcal/mol Å^2^, and only the accommodation of the solvent was allowed. Then, the hydrogen atoms of the protein were freed from the restraint to allow their relaxation. In the third stage, the minimization was done imposing a lighter restriction of 10 kcal/mol Å^2^ on the heavy atoms of the protein. Lastly, a minimization without restraint was conducted. After that, the systems were equilibrated under NVT conditions, heating up from 0 K to 298.15 K in a window of 200 ps and maintaining the final temperature for 100 ps, using the Langevin thermostat with a collision frequency of 2 ps^-1^. Then, the systems were equilibrated for 10 ns under NPT conditions at 298.15 K and 1 atmosphere using the weak-coupling Berendsen barostat. Three production runs of 100 ns under the NPT conditions with random velocities seeds were performed for each protein system. All the MD calculations were performed with periodic boundary conditions with a time step of 2 fs, a 10 Å direct space cutoff for PME and constraining hydrogen atoms with SHAKE algorithm. All the simulations were performed using Amber18 with GPU acceleration (Salomon-Ferrer et al., 2013) and the trajectories were analyzed using *cpptraj* (Roe and Cheatham, 2013).

## Results

### SNP Identification

A set of immune-related genes (*gsto-1*, *ccl19*, *itβ2*, *hsp70*, *mhc-I*) were chosen based on their previous reported role on the immune response in Atlantic salmon (Cepeda et al., 2011; Cepeda et al., 2012; Reyes-López et al., 2015). The identification of SNPs was carried out upon these genes based on data mining analysis. A total of 310 EST sequences obtained from the Unigene database (NCBI) were analyzed to identify SNPs on *ccl19*, *itβ2*, *gsto-1*, *hsp70*, and *mhc-I*. Twenty-eight SNPs were found, broken down into 18 transitions (7 A > G; 11 C > T) and 10 transversions (7 A > C; 1 A > T; 1 C > G; 1 G > T) as the most probable or reliable nucleotide variants (**Table 1**). From them, 3 SNPs were found for *ccl19*, 2 SNPs for *itβ2*, 15 SNPs for *gsto-1*, 4 SNPs for *hsp70*, and 4 SNPs for *mhc-I* (**Table 1**).

**Table 1:** Summary of single-nucleotide polymorphisms (SNPs) identified in a set of immune-related genes expressed in *Salmo salar*.

UniGene ID	Gen	Gene acronym	No. EST included	A > G	C > T	A > C	A > T	C > G	G > T
1668515	*c-c motif chemokine 19 precursor*	*ccl19*	44	1	2	–	–	–	–
3025151	*cd18*	*cd18*	23	1	–	1	–	–	–
1183801	*glutathione transferase omega-1*	*gsto-1*	138	3	7	5	0	0	0
1247008	*heat shock 70 kDa*	*hsp70*	53	1	2	1	–	–	–
3025116	*major histocompatibility complex class I*	*mhc-I*	52	1	–	–	1	1	1

The UniGene ID, number of EST included in the SNP identification per each gene, and the transition (A > G, C > T) and transversion (A > C, A > T, C > G, G > T) nucleotide substitutions are indicated.

### SNP Predicted Functionality

From the nucleotide variations identified, the 21.43% of the SNPs were found in the 5’-UTR region and 53.57% in the 3’-UTR region. At untranslated region level, *mhc-I* showed only nucleotide modifications at 5’-UTR. In the case of *ccl19*, *itβ2,* and *hsp70* genes, the modifications were registered on the 3’-UTR (**Table 2**). Only in the case of *gsto-1* the SNPs were found in both 5’- and 3’-UTR regions. To evaluate the effect of these SNPs located in the UTR regions, the FASTSNP decision tree was used in order to assign a FASTSNP score risk for each of the nucleotide variations found. The results showed that some SNPs on 5’-UTR (*gsto-1*: c.48C > T, c.89A > G; *mhc-I*: c.17G > T) and 3’-UTR (*ccl19*: c.933T > C, c.996C > T; *gsto-1*: c.1068A > C, c.1177A > C; *hsp70*: c.2102C > T, c.2139A > G) obtained the minimum FASTSNP score (FASTSNP score = 0), indicating that these nucleotide variations did not have any predicted consequence. However, the majority of the SNPs detected on these regions had a FASTSNP score of 1–3, which indicates that the SNP generates a low to medium impact (**Table 2**). Based on this SNP functional score effect, the results suggest that the nucleotide variations evaluated could be involved in the regulation of the immune-related genes evaluated.

**Table 2:**
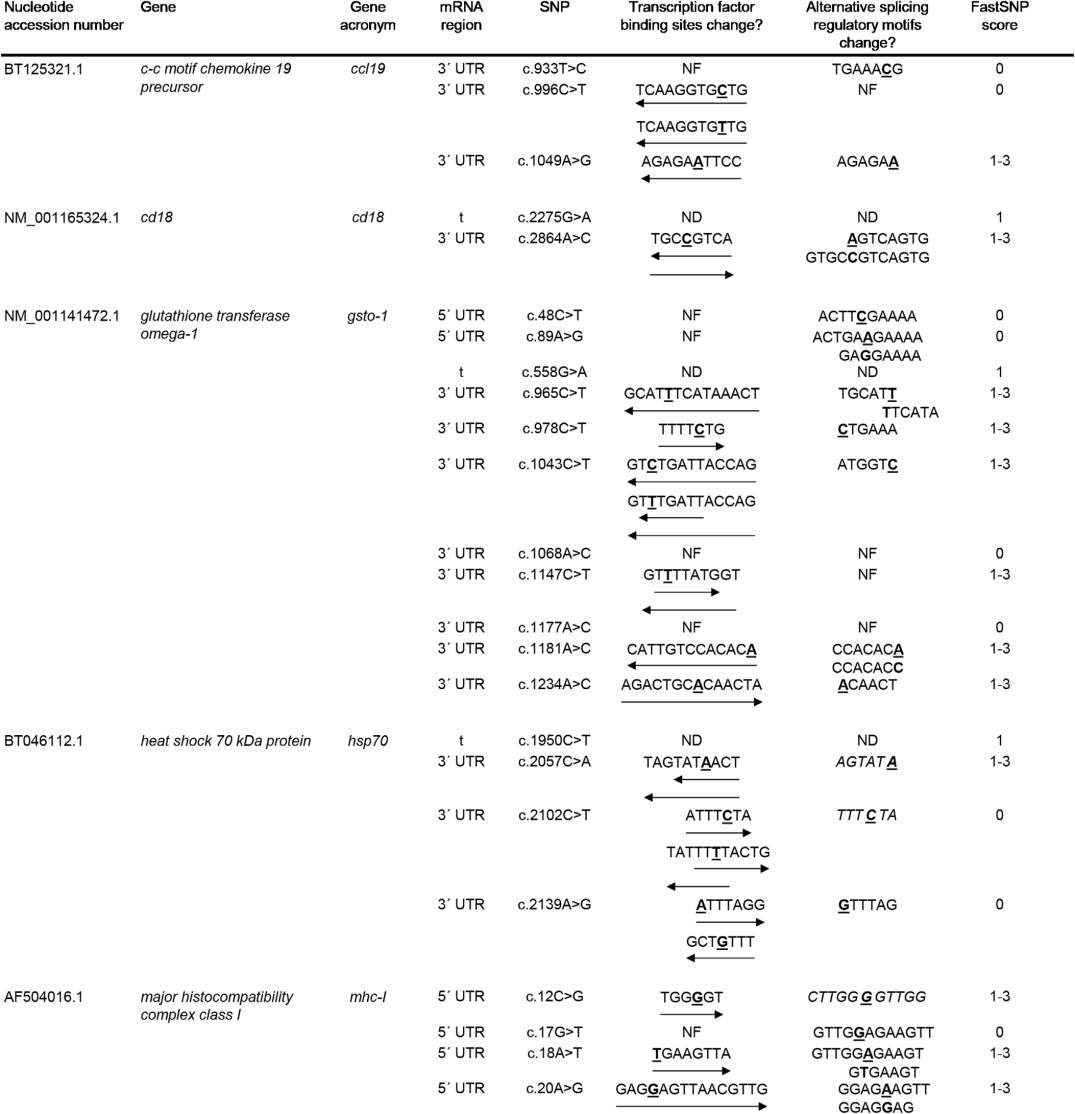
Evaluation of nucleotide variations located in untranslated region (5’-UTR; 3’-UTR) and synonymous single-nucleotide polymorphisms (SNPs) in the translated (t) region on immune-related genes expressed in *Salmo salar*.

Bold and underlined letter represents the nucleotide variation (transcription factor binding site, and alternative splicing regulatory motif). The arrow behind the sequence on the predicted transcription factor binding site column shows the 5’– > 3’ sense and the motif composition. On the alternative splicing regulatory motif column, normal and italic styles indicate the presence of predicted exon splicing enhancer (ESE) or silencer (ESS), respectively. The FASTSNP score was assigned to each SNP (0: minimum score risk; 5: maximum score risk). NF, not found. ND, not determined.

From the total number of SNPs found, the 25% of the nucleotide substitutions were located in the coding region. At functional level, three of them (10.71%) were identified as synonymous SNP since no variation in the predicted amino acid sequence for *itβ2* (c.2275G > A), *gsto-1* (c.558G > A), and *hsp70* (c.1950C > T) was determined. Thus, a low-risk ranking was assigned (FASTSNP score = 1) (**Table 2**). In terms of codon usage, a positive RSCU value was observed for *itβ2* (c.2275G > A) and *gsto-1* (c.558G > A) (**Table 3**). By contrast, *hsp70* (c.1950C > T) showed a negative RSCU value. This antecedents indicate that these sSNPs may have an impact in the local rate of translation elongation. On the other hand, the remaining 14.29% from total nucleotide variations were identified in the coding region as nonsynonymous SNPs. Importantly, all these nucleotide variations were only found into the *gsto-1* sequence (**Table 4**). Two of these variations (c.205A > G, c.484T > C) obtained the highest risk score (SIFT+POLYPHEN score = 5) for nonsynonymous SNPs and whose nucleotide variations represented the modifications in the predicted amino acid sequence of serine (polar, uncharged R group) by glycine (nonpolar, flexible and the smallest R group; S26G) and by proline (nonpolar, cyclic, and rigid R group; S119P), respectively. The other two nucleotide substitutions (c.499T > C, c.769A > C) were ranked in the next risk score level (SIFT+POLYPHEN score = 4), representing in the predicted amino acid sequence a change of tyrosine (polar and aromatic R group) by histidine (polar and sometimes positively charged imidazole R group; Y124H), and threonine (polar, uncharged R group) by proline (T214P), respectively (**Table 4**). Taken together, these results indicate that the SNPs found in the *gsto-1* could have a relevant impact at functional protein structural level.

**Table 3:** Evaluation of relative synonymous codon usage (RSCU) in immune-relevant genes in *Salmo salar*.

Nucleotide accession number	Gene	Gene acronym	sSNP	Amino acid	Codon change		RSCU		ΔRSCU
					From	To	From	To	
NM_001165324.1	*cd18*	*cd18*	c.2275G>A	T	AC**G**	AC**A**	0.48	1.00	0.52
NM_001141472.1	*glutathione transferase omega-1*	*gsto-1*	c.558G>A	A	GC**G**	GC**A**	0.44	0.84	0.40
BT046112.1	*heat shock 70 kDa protein*	*hsp70*	c.1950C>T	P	CC**G**	CC**T**	1.52	1.04	-0.48

Bold letter represents the nucleotide variations in codon for each synonymous single-nucleotide polymorphism (sSNP). RSCU and ΔRSCU values were calculated (ΔRSCU = RSCU mutant - RSCU wild type).

**Table 4:** Effect of nonsynonymous single-nucleotide polymorphisms (SNPs) in glutathione transferase omega-1 coding region of *Salmo salar*.

Nucleotide accession number	Gene	Gene acronym	SNP	Amino acid change	SIFT score	Polyphen score	SIFT+Polyphen score
NM_001141472.1	Glutathione transferase omega-1	*gsto-1*	c.205A>G	S26G	IV	E	5
			c.484T>C	S119P	IV	E	5
			c.499T>C	Y124H	I	D	4
			c.769A>C	T214P	II	D	4

SIFT and POLYPHEN scores are indicated from which the risk is obtained to assess whether the amino acid change produces a variation at protein structural level giving a risk of 1–4, being 1 the higher impact risk. S, serine; G, glycine; P, proline; Y, tyrosine; H, histidine; T, threonine.

### Homology Modeling and All-Atom Explicit Solvent MD Analysis

In order to evaluate whether the predicted SIFT+POLYPHEN score on the nonsynonymous nucleotide variations for *gsto-1* have an impact at the protein structure level, a homology modeling was performed. To do this, the predicted tridimensional protein structure was obtained, on which the effect of such nucleotide variations determined for *gsto-1* were analyzed individually.

Using the CPHModels-3.0 webserver, the higher score alignment (score = 278; E-value = 4e-75) obtained for the *S. salar gsto-1* predicted amino acid sequence was the *Homo sapiens* Glutathione-S-transferase omega 1 (GSTO-1; PDB ID: 1EEM) and thus chosen as the best template structure.

**Figure 1:**
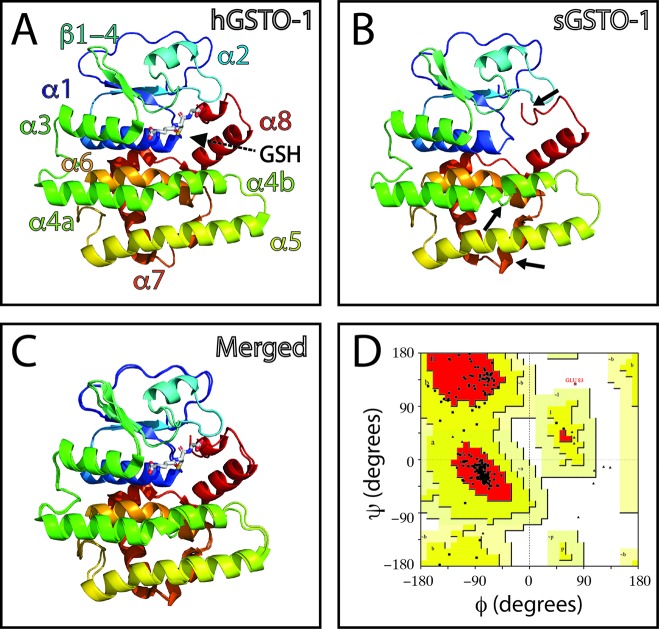
Homology modeling for predicted *S. salar* GSTO-1 based on *H. sapiens* GSTO-1 structure. **(A)** Tridimensional *H. sapiens* GSTO-1 (hGSTO-1) structure. **(B)** Predicted tridimensional *S. salar* GSTO-1 (sGSTO-1) structure. The differences on the secondary structure in the sGSTO-1 compared to hGSTO-1 are shown (arrow lines). **(C)** hGSTO-1 and sGSTO-1 overlay. **(D)** Ramachandran plot for the predicted sGSTO-1 structure. The amino acid distribution into most favored regions (A,B,L), additional allowed regions (a,b,l,p), generously allowed regions (~a,~b,~l,~p), and disallowed regions (GLU83) is indicated. In the protein structures, α-helices (α1- α8), β-sheets (β1-4) are indicated. The reduced glutathione (GSH) molecule is represented.

The overall comparison between the human GSTO-1 structure (**Figure 1A**) and the modeled Atlantic salmon GSTO-1 (**Figure 1B**) showed high similarity, evidencing that the salmon predicted modeled protein does not present serious problems with the structural restrictions dictated by the template. Only some local differences were found by comparing in detail the human and salmon protein model of GSTO-1 at the helixes α4a, α4b, α7, and α8 (**Figures 1B, C**). To further validate the model, a stereochemical evaluation was performed by generating a Ramachandran plot to assess the Phy and Psi dihedral angles distribution. The stereochemical quality of the modeled tridimensional protein structure showed that the amino acids of the predicted sGSTO-1 structure were found mainly within the most favored (89.4%) and the additional allowed (10.1%) energy regions, meanwhile only the 0.5% were at the disallowed regions (**Figure 1D**). This antecedent indicates the good quality of the predicted sGSTO-1 structure obtained.

**Figure 2:**
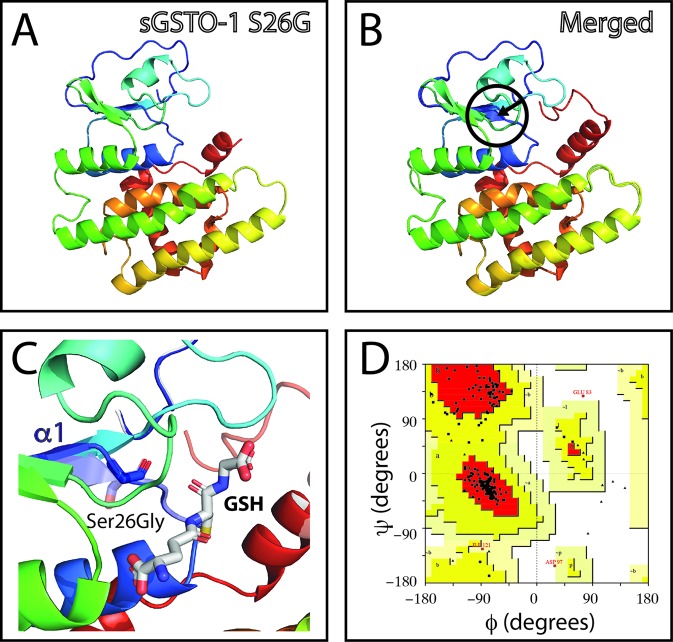
Evaluation of single-nucleotide polymorphism (SNP) c.205A > G effect on the predicted *S. salar* GSTO-1 (sGSTO-1) structure by homology modeling. **(A)** Predicted tridimensional sGSTO-1 including the S26G substitution (sGSTO-1 S26G). **(B)** sGSTO-1 and sGSTO-1 S26P overlay. The punctual amino acid substitution (dotted arrow line) and the region of the secondary structure affected (β2 sheet, circle) are indicated. **(C)** Enlarged view of the sGSTO-1 S26P substitution shown in **(B)**. sGSTO-1 (transparent) and sGSTO-1 (colored) are shown. The reduced glutathione (GSH) molecule is represented. **(D)** Ramachandran plot for the predicted sGSTO-1 S26G structure. The amino acid distribution into most favored regions (A,B,L), additional allowed regions (a,b,l,p), generously allowed regions (~a,~b,~l,~p), and disallowed regions (GLU83, ASP97) is indicated.

The effect of the *gsto-1* nonsynonymous nucleotide variations detected were individually evaluated by homology modeling of *S. salar* GSTO-1. The model of the variant c.205A > G (S26G on the predicted amino acid sequence; sGSTO-1 S26G) suggests that glycine would increase the conformational freedom on the β2 sheet making it shorter (**Figures 2A–C**). The stereochemical quality of the sGSTO-1 S26G protein structure kept the good quality of the model, with only a slight increase in the number of the amino acids in the most favored energy regions (90.3%) compared to the predicted sGSTO-1 structure (**Figure 2D**).

**Figure 3:**
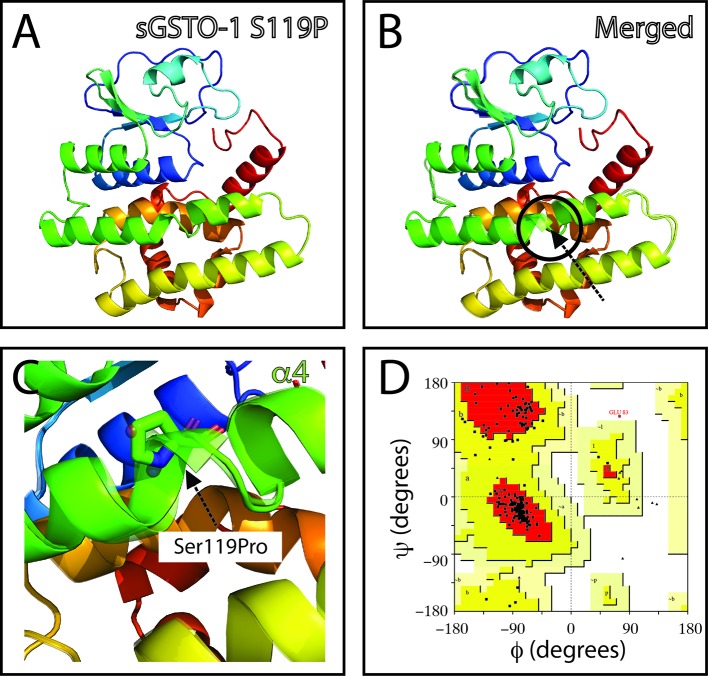
Evaluation of single-nucleotide polymorphism (SNP) c.484T > C effect on the predicted *S. salar* GSTO-1 (sGSTO-1) structure by homology modeling. **(A)** Predicted tridimensional sGSTO-1 including the S119P substitution (sGSTO-1 S119P). **(B)** sGSTO-1 and sGSTO-1 S119P overlay. The punctual amino acid substitution (dotted arrow line) and the structural region (α4 helix kink, circle) are indicated. **(C)** Enlarged view of the sGSTO-1 S119P substitution shown in **(B)**. sGSTO-1 (transparent) and sGSTO-1 (colored) are shown. **(D)** Ramachandran plot for the predicted sGSTO-1 S119P structure. The amino acid distribution into most favored regions (A,B,L), additional allowed regions (a,b,l,p), generously allowed regions (~a,~b,~l,~p), and disallowed regions (GLU83) is indicated.

The SNP c.484T> C (responsible of the modification S119P in the predicted amino acid sequence; sGSTO-1 S119P) is located in the α4 helix kink region (**Figure 3**). The model with the S119P substitution suggests that proline would makes the helix kink longer (**Figures 3B, C**). This modification may generate as consequence that the α4 helix are no longer composed by the α4a and α4b helix but rather independent two different new α-helices. Compared to the sGSTO-1 predicted structure, the sGSTO-1 S119P stereochemical quality showed a slight decrease in the number of the amino acids in the most favored energy regions (87.9%) and an increase on those located in the additional allowed regions (11.6%) compared to the predicted sGSTO-1 structure (**Figure 3D**).

**Figure 4:**
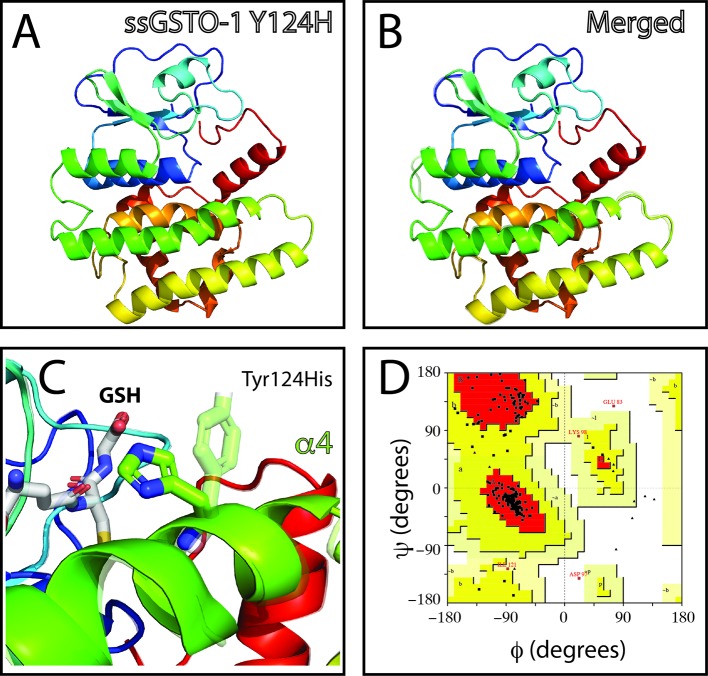
Evaluation of single-nucleotide polymorphism (SNP) c.499T > C effect on the predicted *S. salar* GSTO-1 (sGSTO-1) structure by homology modeling. **(A)** Predicted tridimensional sGSTO-1 including the Y124H substitution (sGSTO-1 Y124H). **(B)** sGSTO-1 and sGSTO-1 Y124H overlay. The punctual amino acid substitution (dotted arrow line) is indicated. **(C)** Enlarged view of the sGSTO-1 Y124H substitution shown in **(B)**. sGSTO-1 (transparent), sGSTO-1 (colored), and the reduced glutathione (GSH) molecule are shown. **(D)** Ramachandran plot for the predicted sGSTO-1 Y124H structure. The amino acid distribution into most favored regions (A,B,L), additional allowed regions (a,b,l,p), generously allowed regions (~a,~b,~l,~p), and disallowed regions (GLU83, ASP97) is represented.

**Figure 5:**
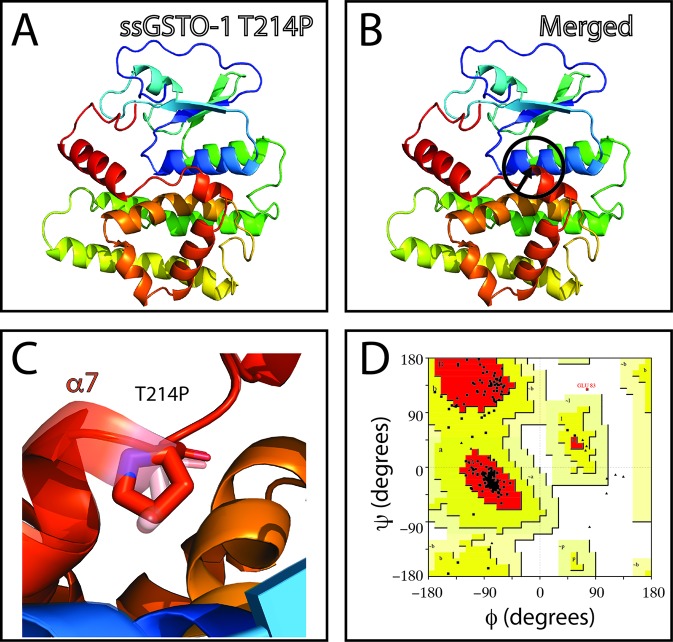
Evaluation of single-nucleotide polymorphism (SNP) c.769A > C effect on the predicted *S. salar* GSTO-1 (sGSTO-1) structure by homology modeling. **(A)** Predicted tridimensional sGSTO-1 including the T214P substitution (sGSTO-1 T214P). **(B)** sGSTO-1 and sGSTO-1 T214P overlay. The punctual amino acid substitution (dotted arrow line) and the region of the secondary structure affected (α7 helix, circle) are indicated. **(C)** Enlarged view of the sGSTO-1 T214P substitution shown in **(B)**. **(D)** Ramachandran plot for the predicted sGSTO-1 structure. The amino acid distribution into most favored regions (A,B,L), additional allowed regions (a,b,l,p), generously allowed regions (~a,~b,~l,~p), and disallowed regions (GLU83) is indicated. In the protein structures, α-helices (α1- α8), β-sheets (β1-4) are indicated. The reduced glutathione (GSH) molecule is represented.

On the other hand, no appreciable variations in the predicted tridimensional *S. salar* GSTO-1 structure were observed for the SNP c499T > C (Y124H substitution; sGSTO-1 Y214H) (**Figure 4**). By contrast, in the case of the SNP c.769C > C (responsible of the modification T214P in the predicted amino acid sequence; sGSTO-1 T214P), a change in the tridimensional conformation affecting the C-terminus region is observed on the α7 helix of the model (**Figure 5**). Punctually, the amino acid substitution makes the α7 helix shorter, thus lengthening the α7 helix kink (**Figures 5B, C**). This region is the special relevance in the protein structure because the residues of the C-terminal end are involved in the formation of the H site, a binding site that accommodates a hydrophobic motif adjacent to the glutathione binding site (Board et al., 2000). The stereochemical quality of the sGSTO-1 T214P was similar to sGSTO-1 (89.9% of the amino acid in the most favored regions) (**Figure 5D**). Thus, the predicted salmon GSTO-1 sequence analysis suggests the high-impact of T214P on the protein functionality.

The predicted protein structure for *S. salar* CCL19 (PDB ID: 2HCI; score = 55; E-value = 2e-08), ITB2 (PDB ID: 2KCN; score = 42; E-value = 2e-04), HSP70 (PDB ID: 1YUW; score = 1036; E-value = 0), and MHC class I (PDB ID: 1KTL; score = 182; E-value = 4e-46) are also shown (**Supplementary Figures 1**–**4**).

**Figure 6:**
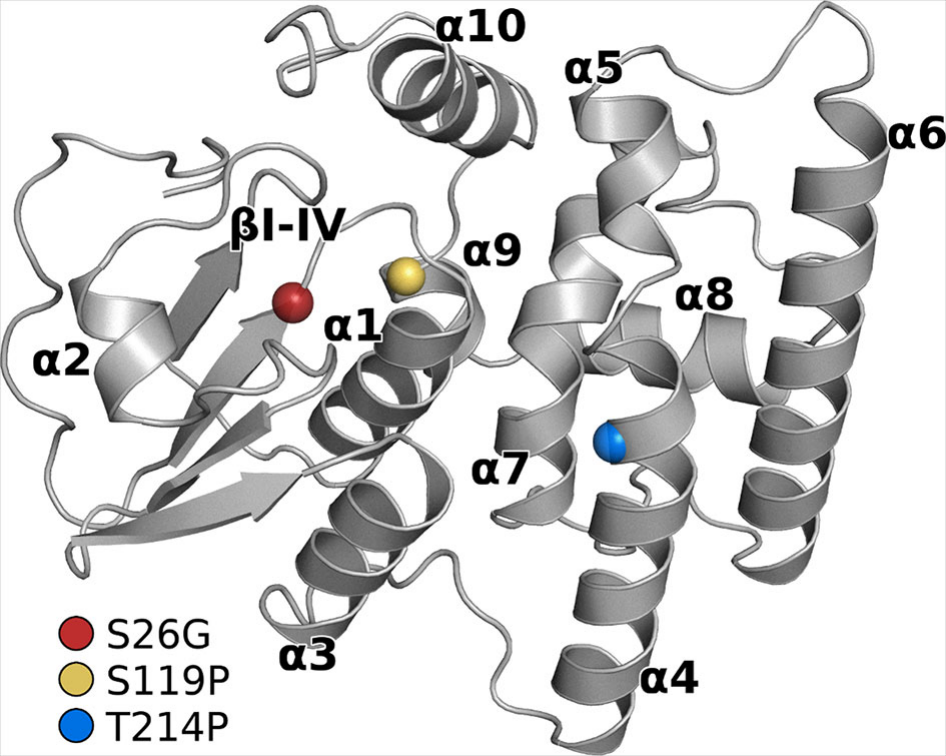
Structure of the GSTO-1 homology model with labeled secondary structure elements. Colored spheres show the position of each punctual variant: red (S26G), yellow (S119P), blue (T214P).

Since homology modeling provides only restricted information about side-chain orientations, three of the GSTO-1 variants that evidenced to be more affected in their structure (S26G; S119P; T214P) were further studied by all-atom MD simulations. Based on that the homology models showed a good stereochemical quality, they were further used as initial coordinates for MD simulations. In **Figure 6** is showed the global structure of the GSTO-1 protein and highlights the location of each mutation evaluated. Analysis of three replicas of 100 ns of simulation for each protein model (GSTO-1 wild-type, S26G; S119P and T214P) showed that the progression of the root mean square deviation (RMSD) of the Cα atoms as a function of time kept values below 3 Å, suggesting they were stable under the time windows explored (**Supplementary Figure 5**).

**Figure 7:**
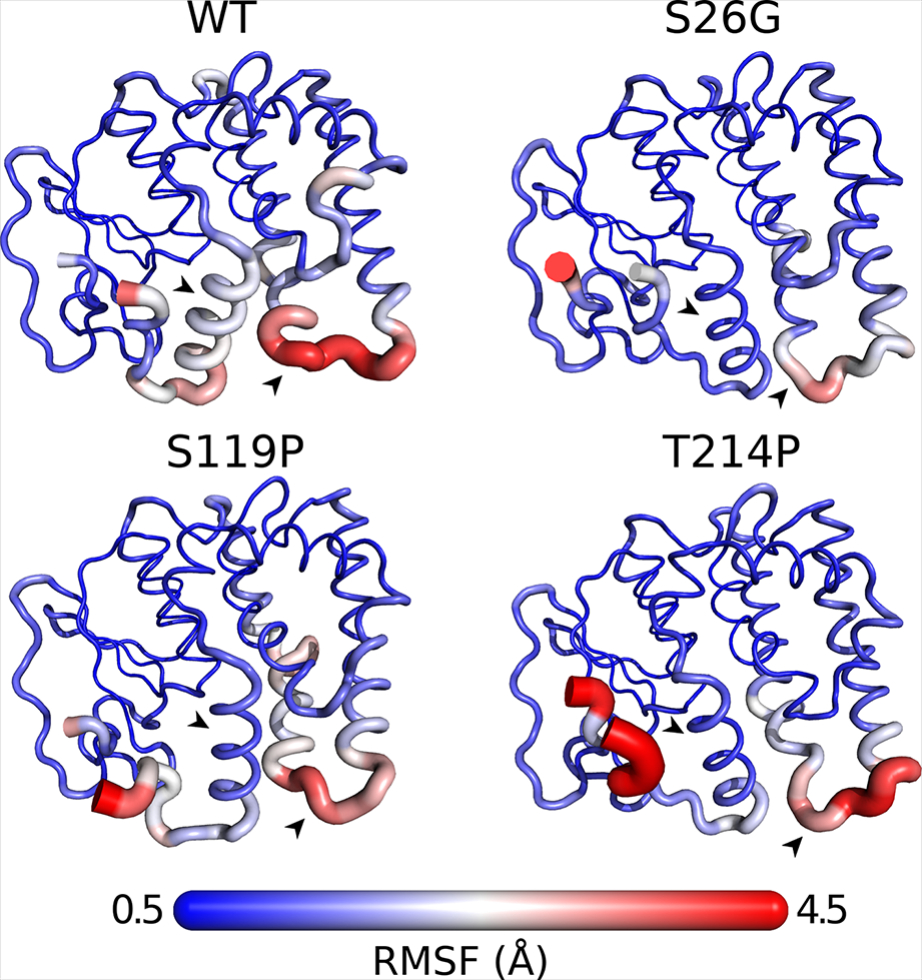
Differences in conformational dynamics between GSTO-1 wild-type (WT), and the variants S26G, S119P, and T214P. The radius of the secondary protein structure and the gradient color (from 0.5 to 4.5 Å) are functions of the RMSF values calculated. As the most affected protein regions, arrowheads highlight the helix α10 (black) and the loop between helixes α6 and α7 (white), respectively.

To assess if the GSTO-1 variants have significant differences in their conformational dynamics, we calculated the root mean square fluctuation (RMSF) over the whole trajectories (300 ns) comparing them with the wild-type trajectories. Importantly, even though the GSTO-1 variants are located far apart within the protein structure, all of them seem to produce similar effects on conformational flexibility (**Figure 7**). Compared to the wild-type GSTO-1 (**Figure 7A**), in the three sGSTO-1 variants explored [S26G (**Figure 7B**), S119P (**Figure 7C**), and T214P (**Figure 7D**)] the α10 helix located at the C-terminus of the protein is notoriously more rigid. Moreover, the loop that connects the helixes α6 and α7 is significantly rigidized in the sGSTO-1 S26G and S119P compared to the wild-type, whereas for the variant sGSTO-1 T214P this loop is only slightly more rigid than the wild-type GSTO-1. The detailed fluctuational profile for each GSTO-1 variant compared to the WT is shown on **Supplementary Figure 6**. Altogether, the differences observed in the local flexibility of secondary structure elements suggests that the SNP variations detected have an impact on the GSTO-1 protein structure and, in consequence, a potential impairment in the protein functionality. Nevertheless, further studies are needed to unravel the precise biophysical consequences of these GSTO-1 variants over the function of the enzyme and their consequences at physiological level.

## Discussion

In this study, we identified a set of SNP located for a group of immune-related genes (*ccl19*, *itβ2*, *gsto-1*, *hsp70*, *mhc-I*) with differential gene expression in *Salmo salar* challenged with IPNV (Cepeda et al., 2011; Cepeda et al., 2012; Reyes-López et al., 2015). These nucleotide variations were localized in both untranslated regions (5’-UTR; 3’-UTR). In the translated region, synonymous (*itβ2*, *gsto-1*, *hsp70*) and nonsynonymous (*gsto-1*) SNPs were found. The potential impact of the nonsynonymous SNPs was also evaluated based on the predicted tridimensional *S. salar* GSTO-1 structure obtained through homology modeling. To our knowledge, currently there are no previous studies in Atlantic salmon in which the SNP functional effect had been evaluated at predicted tridimensional protein structure level. These results open the possibility that these candidate SNPs found on genes with immune function could be associated to the inter-individual gene expression variability in Atlantic salmon and their response against pathogens they are exposed at aquaculture rearing conditions.

The interest in identifying SNPs is given by the large amount of information that exists in model organisms that relate these nucleotide variations with susceptibility to diseases (Mooney et al., 2010), which may allow to identify a series of candidate genes as targets for therapies (Voisey and Morris, 2008). In salmon, the association between polymorphism and susceptibility has been suggested (Correa et al., 2017; Holborn et al., 2018). For this reason, the identification of polymorphisms including SNPs in genes of immunological relevance may help to establish associations between polymorphism and susceptibility to diseases affecting the productive sector. In this way, the identification of SNPs and its analysis through the use of different bioinformatics resources (including those intended for the evaluation of risk scores and estimating its consequences in the predicted three-dimensional protein structure from the predicted amino acid sequence) appears as an interesting alternative to select more accurately those candidate SNPs from a set of SNPs identified with a predicted impact.

Different strategies have been used to identify SNP including small (single-strand conformation polymorphism (SSCP), heteroduplex analyses, random shotgun, polymerase chain reaction (PCR) product sequencing, EST analysis) (Liu and Cordes, 2004), and high scale strategies (next generation sequencing (NGS) and whole genome sequencing) (Abdelrahman et al., 2017; Kumar and Kocour, 2017). Particularly, the EST sequence analysis-based SNP identification is not new although it has not been extensively explored. In this ambit, the EST sequences available on data repositories allow to exploit the redundancy existent in these collections making feasible the analysis on transcripts of the same gene from multiple individuals to identify potential true SNPs minimizing the sequencing errors (Hayes et al., 2007a). Such volume of information becomes an excellent database for the search of candidate polymorphisms through the use of bioinformatics approaches (Irizarry et al., 2000; Guryev et al., 2004). In Atlantic salmon previous reports have been carried out to identify SNPs based on ESTs data (Hayes et al., 2007b) with a high percentage (72.4%) of successfully validated SNPs from the putative identified SNPs obtained (Hayes et al., 2007a). Altogether, the search of SNP from the EST database in Atlantic salmon could contribute to the identification of a set of candidate SNP with a high percentage of success, and also allows to extract the maximum possible benefit from the large amount (and often little exploited) of available information in the data repositories. The use of integrated bioinformatics resources to evaluate the potential consequences of those SNPs (in terms of risk factor and predicted protein three-dimensional structure) could favor the selection of certain candidate SNPs for their validation (both genetic and functional) as well as could shed lights of their effects from a physiological context point of view.

According to Hayes et al. (Hayes et al., 2007b), our results showed a higher number of transition (A > G, C > T) than transversion (A > C, A > T, C > G, G > T) nucleotide substitutions. At the same time, our results showed that 25% of the functional nucleotide variants were associated to synonymous and nonsynonymous modifications. Importantly, a previous study reported a total of 23.8% of the SNPs found with these functional variations (Hayes et al., 2007b). These antecedents suggest that the methodology used in this study may result in the identification of a set of candidate SNPs using *in silico* approaches based on information that is currently contained from public databases.

Previous reports have assessed the use of the bioinformatics tools used in our current study (Mohamed et al., 2008; Capasso et al., 2009; Liu et al., 2009; Wei et al., 2010; Rajasekaran and Sethumadhavan, 2010). Several reported have used FASTSNP as a tool to evaluate the SNP significance in genes associated to disease susceptibility in mammals (Ali Mohamoud et al., 2014; Panda and Suresh, 2014; Phani et al., 2014; Liu et al., 2016; Yu et al., 2017). For SNP identification, we used the FASTSNP decision tree to evaluate whether the nucleotide variation on noncoding region for *ccl19*, *itβ2*, *gsto-1*, *hsp70*, and *mhc-I* could have an predictive effect over gene processes including transcription binding sites or splicing regulation (Yuan et al., 2006). Most of these nucleotide substitutions were ranked with a score 1-3, assigned by the predictive change upon transcription factor binding sites. Furthermore, changes affecting predicted exonic splicing enhancer or exonic splicing silencer motif may also compromise the gene function (Fairbrother et al., 2002; Cartegni et al., 2003; Fairbrother et al., 2004; Wang et al., 2004). Importantly, the link between alternative splicing on immune-related genes in Atlantic salmon has been previously proposed using IPNV (Maisey et al., 2011), suggesting that SNPs affecting these regulatory processes could play a relevant role on the host immune response. Alterations in the immune response associated to SNP located on untranslated regions have been reported in mammals including *ccl19* (Muinos-Gimeno et al., 2009; Elton et al., 2010; Cai et al., 2014), *itβ2* (Bachmann et al., 1997; Mueller et al., 2004; Zhao et al., 2013), and *mhc-I* (Cree et al., 2010). By contrast, in teleost there is a clear lack of information about polymorphisms located on the coding region of these genes, being only reported previously on *hsp70* (*Miichthys miiuy*) and particularly in *mhc-I* of Atlantic salmon (Grimholt et al., 2002; Grimholt et al., 2003; Miller et al., 2004; Glover et al., 2007; Wynne et al., 2007), probably because its relevant role on the antigen presentation and the subsequent immune response activation. In regard to the modulation of these genes on Atlantic salmon against pathogens, in IPNV-infected salmon the expression of all the genes evaluated in this current study have been identified and annotated from splenic leukocytes cDNA library obtained from IPNV-infected Atlantic salmon (Cepeda et al., 2011; Cepeda et al., 2012). In addition, the expression for *ccl19*, *itβ2*, *hsp70*, and *mhc-I* was modulated in head kidney Atlantic salmon full-sibling families (Reyes-López et al., 2015). In the case of *ccl19*, its expression is upregulated in trout head kidney leucocytes infected with IPNV (Montero et al., 2009), suggesting a role in inflammation and immune response mediated by the recruitment of lymphocytes (Reyes-López et al., 2015). In the same study, we detected a differential expression pattern between IPN-susceptible and IPN-resistant families in the expression of *ccl19* and *itβ2* (*cd18*), noting in the susceptible families an abrupt high expression at 1 day post-infection (dpi) to then drop drastically at 5 dpi, meanwhile the expression in the IPN-resistant families remained constant (Reyes-López et al., 2015). In the case of *hsp70* and *mhc-I* a similar upregulation was observed in the susceptible and resistant families to IPNV, but differences were found at 5 dpi when expression dropped to basal levels in susceptible families but not in those resistant, suggesting that an impaired antigen presentation can contribute to the IPN-susceptible phenotype of salmon (Reyes-López et al., 2015). Further studies are needed to elucidate if there is a correlation between the candidate SNPs found from the SNP mining analysis on this current study and IPN-resistance.

Synonymous SNPs have been often called “silent” SNPs because they do not induce a change in the protein amino acid composition due to the degenerating genetic code (more than one codon translate the same amino acid). In the last years, sSNPs are attracting more attention in mammals since these silent mutations appear to be linked to a large list of diseases (Sauna and Kimchi-Sarfaty, 2011). However, in fish no attention has been paid to these mutations so far. In human, these sSNPs can lead to disease by different mechanisms: disrupting splicing signals (Parmley and Hurst, 2007) altering regulatory binding sites (Stergachis et al., 2013); and by changing the secondary structure of the mRNA affecting protein expression (Brest et al., 2011). It has been also described that a change in the codon usage may compromise the translation elongation by introducing a rarer codon (associated to a negative ΔRSCU value). This leads to the use of a less abundant tRNA that may decrease the rate of local elongation and, in consequence, a lower protein synthesis levels and/or protein missfolding. By contrast, the opposite above-mentioned effect may take place in those nucleotide variants with a positive ΔRSCU value (Sauna and Kimchi-Sarfaty, 2011). In our study, one negative ΔRSCU value was assign (*hsp70* c.1950C > T), and two positive ΔRSCU values (*itβ2* c.2275G > A; *gsto-1* c.558G > A). These values suggest that these sSNPs may be associated to differences in the local rate of elongation. Nonetheless, it is not clear if these nucleotide variants may cause a change at physiological level (Sauna and Kimchi-Sarfaty, 2011). Further studies are needed to elucidate the physiological consequences of these polymorphisms.

We obtained the score for the nonsynonymous SNPs based on the cutoffs for SIFT and POLYPHEN because they have previously deemed appropriate for those nucleotide variations that in mammals may play a role on infective processes (Xi et al., 2004; Bhatti et al., 2006). The ranking scheme strategy used in our study has the purpose to evaluate *in silico* the nucleotide variations on sequences of interest in order to provide semiquantitative information and primarily intended for its use in the absence of biochemical characterization. The nonsynonymous SNPs were found only in the *gsto-1* gene sequence. GSTO-1 is an enzyme involved in biotransformation of compounds including toxic substances and oxidative stress products, transport of ligands, and regulation of signaling pathways (Burmeister et al., 2008). The GSTO-1 enzyme is responsible for catalyzing the reaction that results from glutathione conjugates generated by GSH in response to damage signals, thus influencing the relationship between reduced glutathione and oxidized glutathione (GSH/GSSG) modulating the expression of cytokines related to the Th1 immune response (Dobashi et al., 2001), thus playing a key role on the host immune response modulation. To date, very few studies have identified the effect of SNP into the *gsto-1* sequence (He et al., 2013; Zmorzynski et al., 2015), including teleost species (Liao et al., 2017). The analysis of the four *gsto-1* nonsynonymous SNPs found by SIFT and POLYPHEN showed two of them with the second (c.205A > G; c484T > C) and the highest (c.499T > C; c.769A > C) score possible. These results indicate that the protein would be affected on its tridimensional structure.

Despite the relevance and progress made on the SNP identification, there is a clear lack of studies in which would be proposed predictive models that could help to visualize the consequence of those SNPs located on the translated region and ranked with a high-risk score. In fact, one of the limitations of these tools is there is no reliable proposal about the changes that could take place in the protein structure. Hence, it is not possible to estimate at the structural level if the high impact score for the nucleotide modifications evaluated may have an effect in essential sectors for the protein functionality such as the active site or other fundamental interactions involved in the stabilization of its structure. To carry out this kind of analysis is necessary to characterize the mutant *in vitro* and determine the protein structure by, for instance, X-ray crystallography. Regrettably, the knowledge about salmon tridimensional protein structures is still very limited, with no protein structural information for GSTO-1. Therefore, in order to evaluate the nonsynonymous SNP effect at protein structure level it is strictly necessary the generation of the predicted tridimensional protein structure using bioinformatics tools. Thus, the homology modeling strategy could be used to predict the tridimensional structure of the protein and, with it, to be able to establish a relationship between the nonsynonymous SNPs-provoked structural protein changes and the relevance of them (SIFT+POLYPHEN score).

Although there is no previous antecedents in fish, the SNP effect on the protein structure has been evaluated on genes of immunologic interest (Jang et al., 2017; Majumdar et al., 2017; Melzer and Palanisamy, 2018), including human glutathione-S-transferase superfamily (Kitteringham et al., 2007). The structure of GSTO-1 is composed mainly of an N-terminal domain of the thioredoxin type which consists of four central β-sheets surrounded at each end by α-helices, and a C-terminal end which is mainly made of α-helices (Board et al., 2000). The latter helices, fold over the N-terminal domain generating a network of hydrogen bonds which define a continuous surface (β/α structure) (Board et al., 2000). This β/α structure could be destabilized by the SNP c.205A > G (S26 amino acid substitution) located in β2 sheet, which could introduce a structural conformational freedom either in the β1 sheet as in the adjacent one (β2 sheet). In addition, on the N-terminal end there is the glutathione binding site, also called G-site, in which the cysteine 32 (located at the N-terminal end of the α1 helix) forms a disulfide bond in the presence of reduced glutathione (GSH) (Rossjohn et al., 1998). It has been described in other GST proteins that a H-bond between the GSH sulfur and the OH of the tyrosine or serine stabilizes the GSH thiolate anion (Kortemme and Creighton, 1995). Mutations of these serine or tyrosine sites lead to a substantial or complete protein inactivation (Stenberg et al., 1991; Board et al., 1995). Based on the predicted tridimensional *S. salar* GSTO-1, the S26 is located in the proximity of the G-site. Taken together, the S26G substitution could affect the protein functionality, although more studies are needed to establish the consequence at protein level of this candidate nonsynonymous SNP.

The predicted protein structure for sGSTO-1 S119P showed a modification on the α4 helix kink region, rendering it longer compared with the wild-type. This probably because the proline substitution prevents the H-bonds network within the alpha helix. In a previous report has been described that proline modifies the secondary protein structure, suggesting that the replacement of Ser119Pro probably interferes with the α4 helix formation (Chiu et al., 2013). On the other hand, despite the high SIFT+POLYPHEN score obtained for the SNP c.499T > C, no appreciable changes in terms of the secondary structure were observed in the predicted sGSTO-1 Y124H model. However, the physicochemical change may have impacts that are not easily predicted from a structural point of view.

The homology model for the SNP c.769A > C (sGSTO-1 T214P) showed a change in the tridimensional conformation at the C-terminal end of the α7 helix of the protein. The α7 helix is directly involved in the H site of the protein, which has a hydrophobic motif and is adjacent to the G binding site. This site is composed of both the N-terminal and the C-terminal ends and has the putative function of being a binding site for GSH or other target molecules. In the sGSTO-1, the threonine 214 (hydrophilic nature) forms part of a hydrophobic motif, contributing to the formation of H bonds, thus allowing the union of a greater variety of substrates (Board et al., 2000). Therefore, the T214 substitution could decrease the efficiency of the protein, since having an exclusively apolar character decreases the range of substrates that can bind to this active site.

MD simulations of those SNPs with a relevant impact based on the predicted GSTO-1 structure (S26G; S119P; T214P) were also assessed. The results show that these variations affect the dynamics of common regions within the GSTO-1 structure, turning the overall structure of the enzyme more rigid than the wild-type variant. From the three variants above-mentioned assayed, only T214P is located directly in the main affected helix, while the other two (S26G; S119P) are located closer to the N-terminal region. Since the protein structure is a complex network of covalent and noncovalent interactions, the result obtained by MD simulations implies that the individual local effect of each one of these substitutions is being transduced throughout the protein structure and affecting regions that are likely to be determining in the stability or the catalytic capabilities of the enzyme. Since conformational dynamics are an essential aspect of a protein function, these differences are probably impacting the functioning of sGSTO-1, and consequently the fitness of the organism (Bhabha et al., 2013). Enhanced rigidity is a trait commonly associated with more stable proteins (Radestock and Gohlke, 2011). Therefore, it is possible that these SNPs could alter the stability of GSTO-1. On the other hand, although these variations are not located at the binding site of the enzyme, it is possible that these SNPs could have allosteric effects modulating the catalysis. This because conformational dynamics can play a role in multiple aspects of enzymatic activity like accessibility of the substrate to the active site, product release rate and the probability of finding catalytically active populations within the conformational ensemble (Henzler-Wildman et al., 2007; Kokkinidis et al., 2012).

Altogether, and based on the modeled sGSTO-1 structure, most of the candidate nonsynonymous SNP identified showed changed in the secondary structure dynamics. Therefore, the strategy to evaluate nonsynonymous SNP based on homology modeling and all-atom MD simulations provide additional evidence that may help to rank them in order to subsequently validate those that are most relevant according to their structural effect. Further studies should be focused in the *in vitro* characterization of these enzymes to generate a complete biophysical picture of the effects of these SNPs in these genes associated to immune function.

## Author Contributions

The conceptualization of the study was performed by EV-V, MI, and FER-L. The methodology was originally proposed by EV-V, KM, AMS, MI, and FER-L. The SNP search and identification was carried out by EV-V, SR-C, JY, and FER-L. The codon usage analysis was carried out by EV-V, HV, and FER-L. The homology modeling was conducted by EV-V, JAR-P, KM, and FER-L. The molecular dynamics simulations were performed by JAR-P, PC, VC-F, and FER-L. EV-V, SR-C, JAR-P, KM, JY, HV, PC, VC-F, LT, AMS, MI, and FER-L participated actively in the data analysis and interpretation. EV-V, SR-C, and FER-L wrote the original draft. All the authors corrected, read, and approved the final manuscript.

## Funding

This study was supported by INNOVA-CORFO (No. 09MCSS-6691 and 09MCSS-6698), FONDECYT (No. 1161015; 11150807; 11180705; 11181133), DICYT- USACH, VRIDEI-USACH (USA1899 VRIDEI 021943IB-PAP), and Universidad Mayor startup funds (No. OI101205; SR-C). The authors also thank to the grants from CONICYT-BCH (Chile) Postdoctoral fellowship (No. 74170091; EV-V), International postdoctoral stay 2019 (Universidad de Chile, UCH1566; EV-V) and VRIDEI-USACH (FR-L). The funders had no role in study design, data collection and analysis, decision to publish, or preparation of the manuscript.

## Conflict of Interest

The authors declare that the research was conducted in the absence of any commercial or financial relationships that could be construed as a potential conflict of interest.

## Supplementary Material

Figure S1 Homology modeling for predicted *S. salar* CCL19 based on tridimensional *H. sapiens* CCL19 structure. **(A)** Tridimensional *H. sapiens* CCL19 (hCCL19) structure. **(B)** Predicted tridimensional *S. salar* CCL19 (sCCL19) structure. **(C)** hCCL19 and sCCL19 overlay. **(D)** Ramachandran plot for the predicted sCCL19 structure. The amino acid distribution into most favored regions (A,B,L), additional allowed regions (a,b,l,p), generously allowed regions (~a,~b,~l,~p) is indicated.

Figure S2 Homology modeling for predicted *S. salar* ITB2 based on tridimensional *H. sapiens* ITB2 structure. **(A)** Tridimensional *H. sapiens* ITB2 (hITB2) structure. **(B)** Predicted tridimensional *S. salar* ITB2 (sITB2) structure. **(C)** hITB2 and sITB2 overlay. **(D)** Ramachandran plot for the predicted sITB2 structure. The amino acid distribution into most favored regions (A,B,L), additional allowed regions (a,b,l,p), generously allowed regions (~a,~b,~l,~p) is represented.

Figure S3 Homology modeling for predicted *S. salar* HSP70 based on tridimensional *H. sapiens* HSP70 structure. **(A)** Tridimensional *H. sapiens* HSP70 (hHSP70) structure. **(B)** Predicted tridimensional *S. salar* HSP70 (sHSP70) structure. **(C)** hHSP70 and sHSP70 overlay. **(D)** Ramachandran plot for the predicted sHSP70 structure. The amino acid distribution into most favored regions (A,B,L), additional allowed regions (a,b,l,p), generously allowed regions (~a,~b,~l,~p) and disallowed regions (GLN83 and ALA540) is represented.

Figure S4 Homology modeling for predicted *S. salar* MHC class I based on tridimensional *H. sapiens* MHC class I structure. **(A)** Tridimensional *H. sapiens* MHC class I (hMHC class I) structure. **(B)** Predicted tridimensional *S. salar* MHC class I (sMHC class I) structure. **(C)** hMHC class I and sMHC class I overlay. **(D)** Ramachandran plot for the predicted sMHC class I structure. The amino acid distribution into most favored regions (A,B,L), additional allowed regions (a,b,l,p), generously allowed regions (~a,~b,~l,~p) and disallowed regions (ASP39 and ILE40) is represented.

Figure S5 Molecular dynamics simulation for the wild-type GSTO-1 **(A)** and the variants S26G **(B)**, S119P **(C)**, and T214P **(D)**. The root mean square deviation (RMSD) of the Cα atoms on a time windows explored of 100 ns is shown for each replicate.

Figure S6 Root mean square fluctuation (RMSF) profiles for each mutant analyzed (S26G; S119P; T214P) compared to the GSTO-1 wild-type.
