# Supplementary figures and images for: Single-Nucleotide Polymorphisms (SNP) Mining and Their Effect on the Tridimensional Protein Structure Prediction in a Set of Immunity-Related Expressed Sequence Tags (EST) in Atlantic Salmon (Salmo salar)
Source: Front Genet. 2020 Feb 27;10:1406. doi: 10.3389/fgene.2019.01406 (PMC7056891; doi:10.3389/fgene.2019.01406)

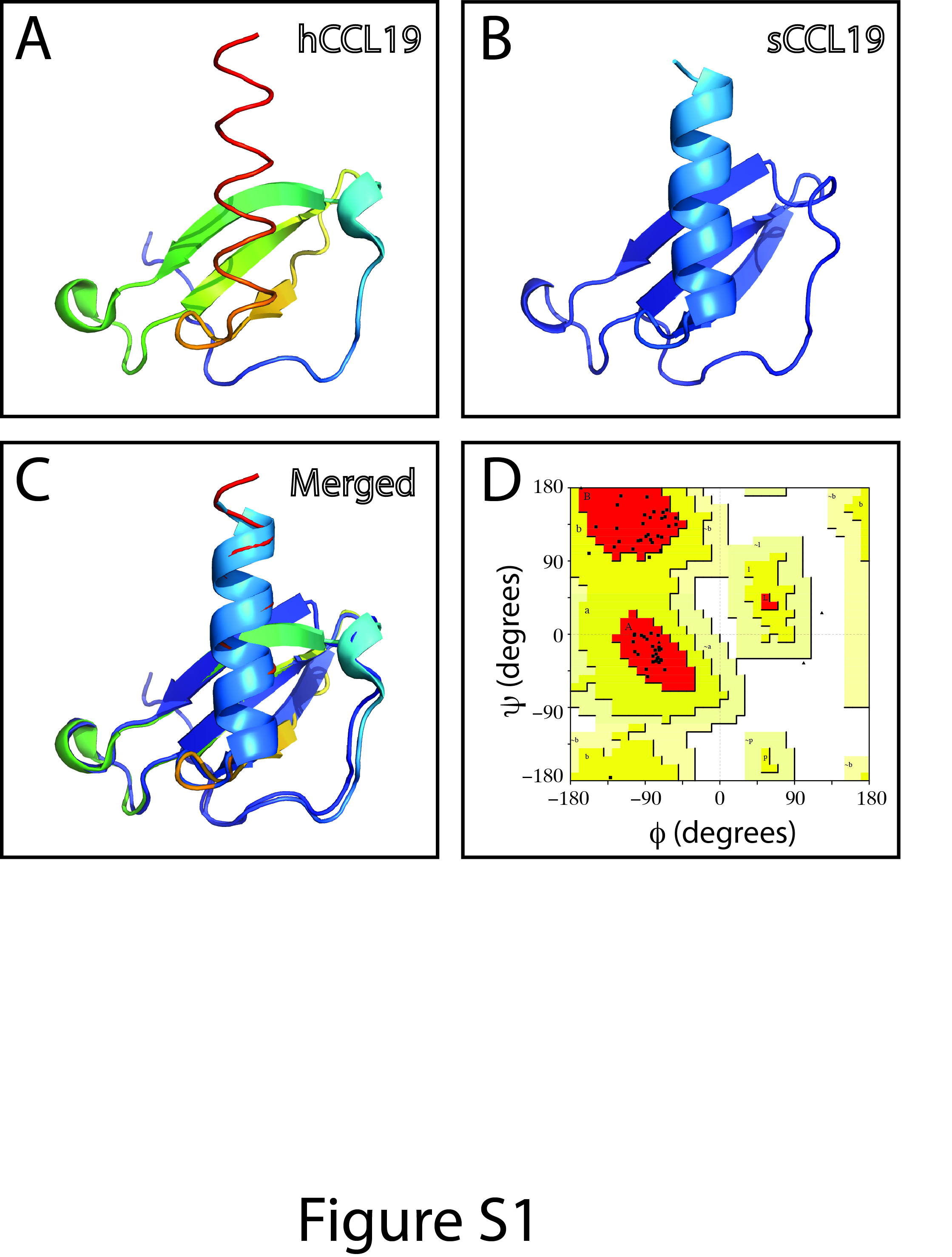

Supplement: Figure S1 — Homology modeling for predicted S. salar CCL19 based on tridimensional H. sapiens CCL19 structure. (A) Tridimensional H. sapiens CCL19 (hCCL19) structure. (B) Predicted tridimensional S. salar CCL19 (sCCL19) structure. (C) hCCL19 and sCCL19 overlay. (D) Ramachandran plot for the predicted sCCL19 structure. The amino acid distribution into most favored regions (A,B,L), additional allowed regions (a,b,l,p), generously allowed regions (~a,~b,~l,~p) is indicated. [file Image_1.tif]

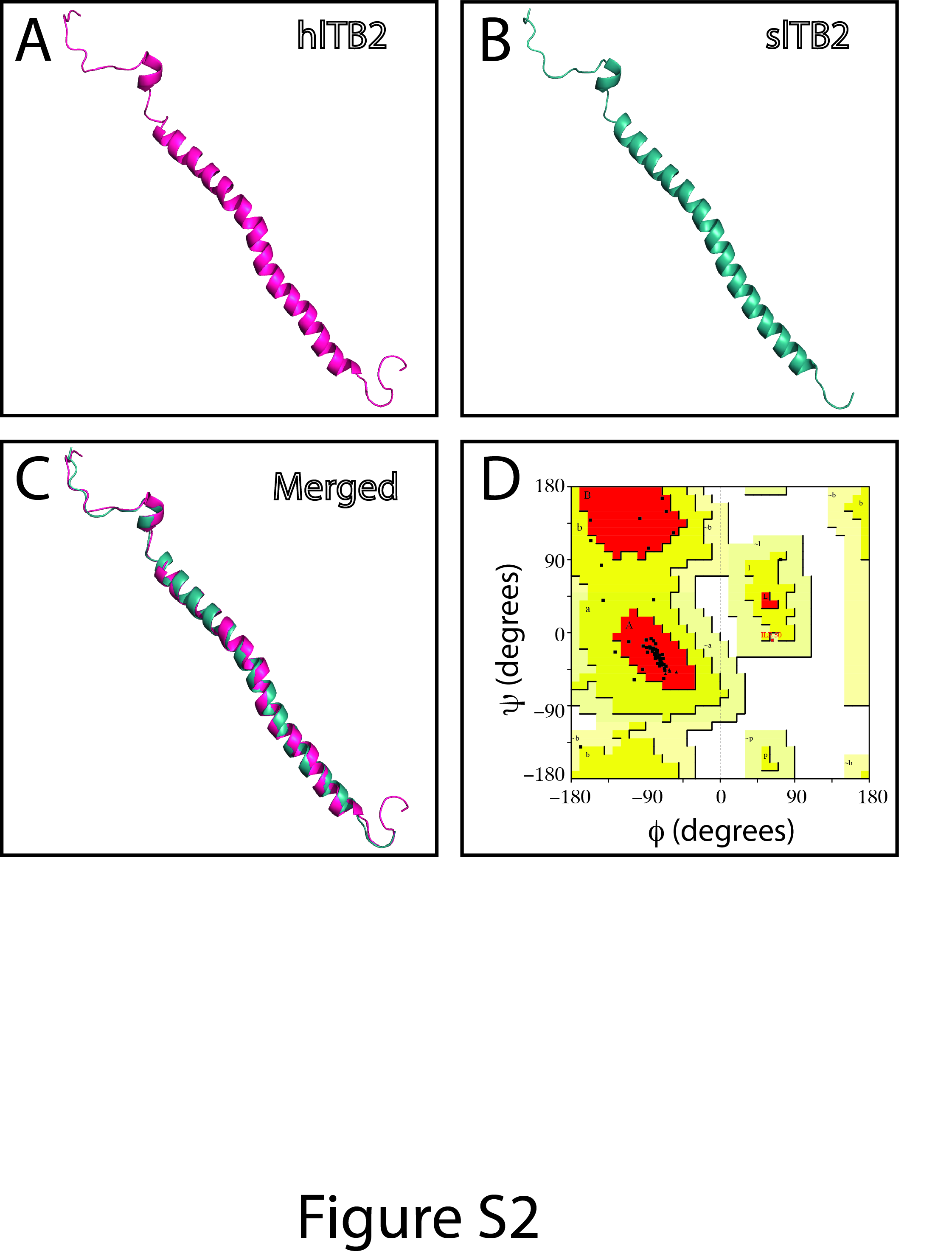

Supplement: Figure S2 — Homology modeling for predicted S. salar ITB2 based on tridimensional H. sapiens ITB2 structure. (A) Tridimensional H. sapiens ITB2 (hITB2) structure. (B) Predicted tridimensional S. salar ITB2 (sITB2) structure. (C) hITB2 and sITB2 overlay. (D) Ramachandran plot for the predicted sITB2 structure. The amino acid distribution into most favored regions (A,B,L), additional allowed regions (a,b,l,p), generously allowed regions (~a,~b,~l,~p) is represented. [file Image_2.tif]

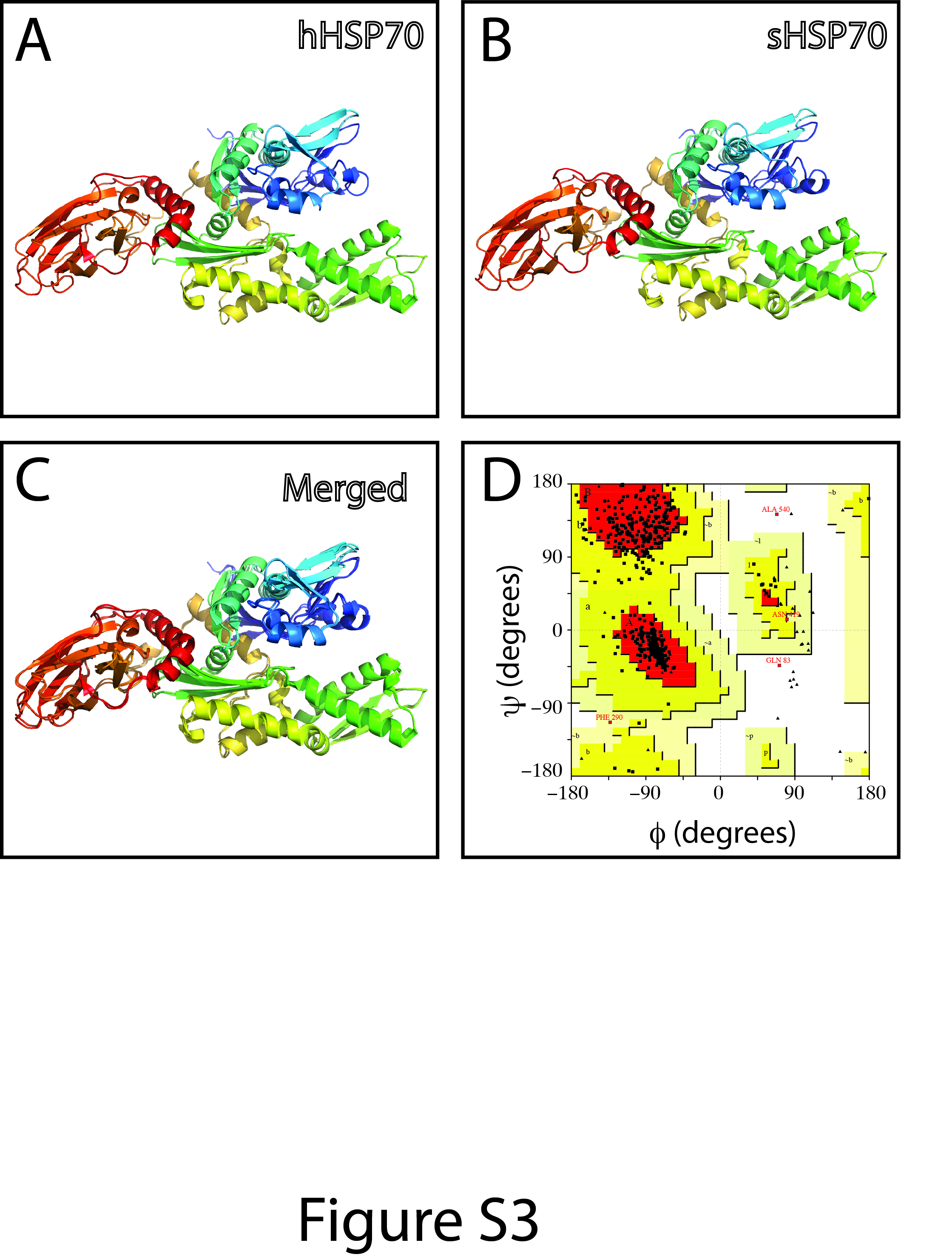

Supplement: Figure S3 — Homology modeling for predicted S. salar HSP70 based on tridimensional H. sapiens HSP70 structure. (A) Tridimensional H. sapiens HSP70 (hHSP70) structure. (B) Predicted tridimensional S. salar HSP70 (sHSP70) structure. (C) hHSP70 and sHSP70 overlay. (D) Ramachandran plot for the predicted sHSP70 structure. The amino acid distribution into most favored regions (A,B,L), additional allowed regions (a,b,l,p), generously allowed regions (~a,~b,~l,~p) and disallowed regions (GLN83 and ALA540) is represented. [file Image_3.tif]

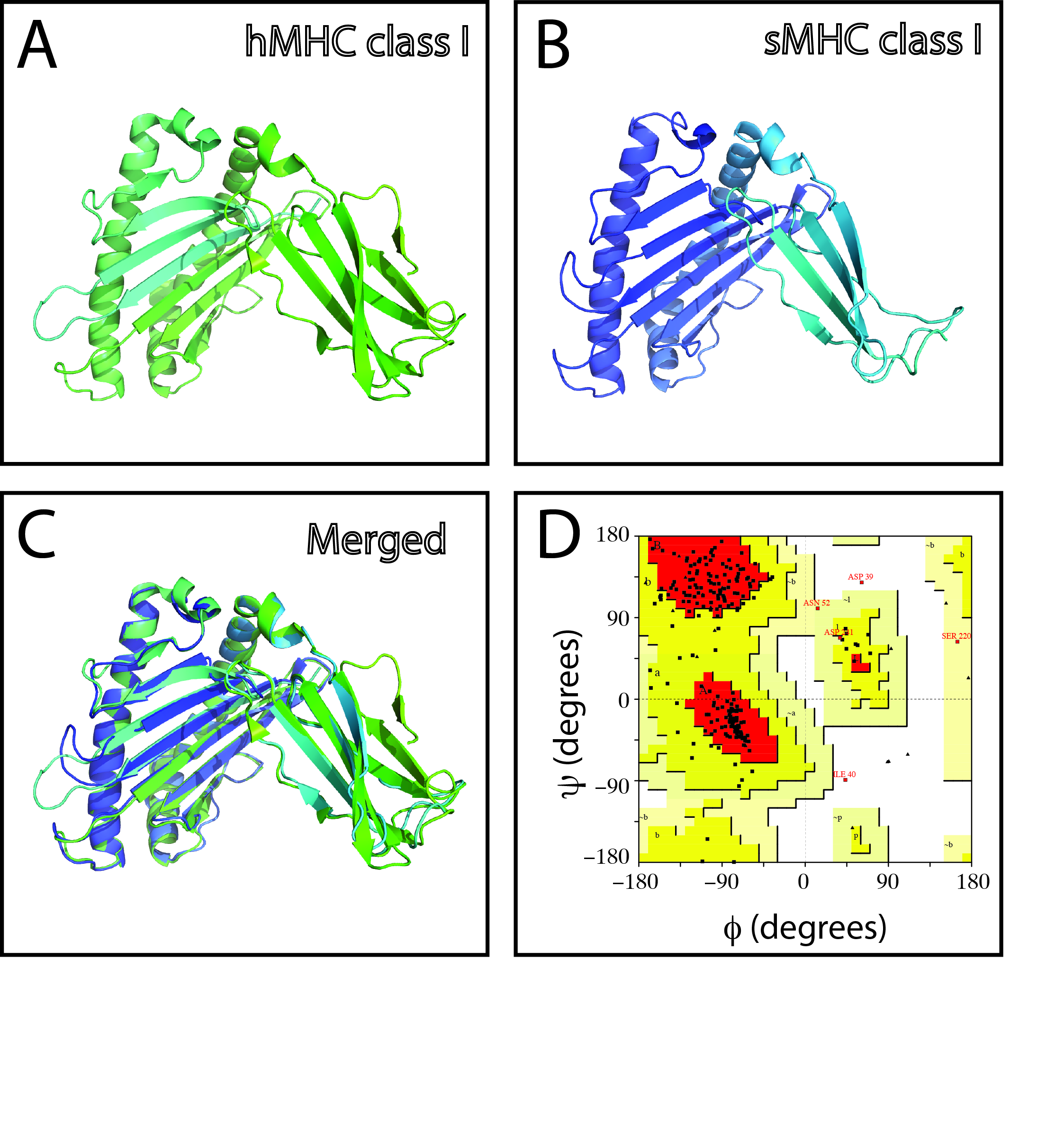

Supplement: Figure S4 — Homology modeling for predicted S. salar MHC class I based on tridimensional H. sapiens MHC class I structure. (A) Tridimensional H. sapiens MHC class I (hMHC class I) structure. (B) Predicted tridimensional S. salar MHC class I (sMHC class I) structure. (C) hMHC class I and sMHC class I overlay. (D) Ramachandran plot for the predicted sMHC class I structure. The amino acid distribution into most favored regions (A,B,L), additional allowed regions (a,b,l,p), generously allowed regions (~a,~b,~l,~p) and disallowed regions (ASP39 and ILE40) is represented. [file Image_4.tif]

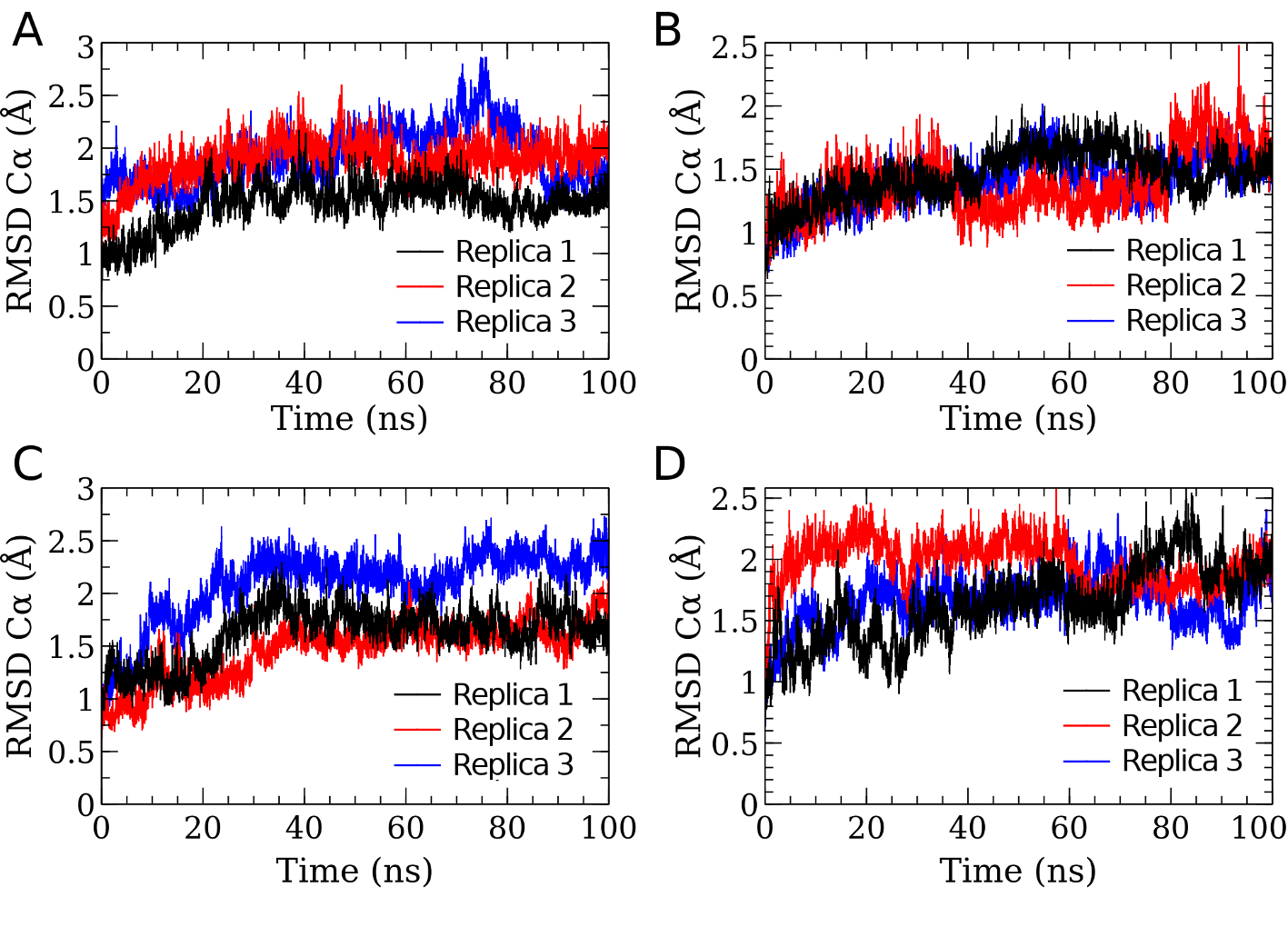

Supplement: Figure S5 — Molecular dynamics simulation for the wild-type GSTO-1 (A) and the variants S26G (B), S119P (C), and T214P (D). The root mean square deviation (RMSD) of the Cα atoms on a time windows explored of 100 ns is shown for each replicate. [file Image_5.tiff]

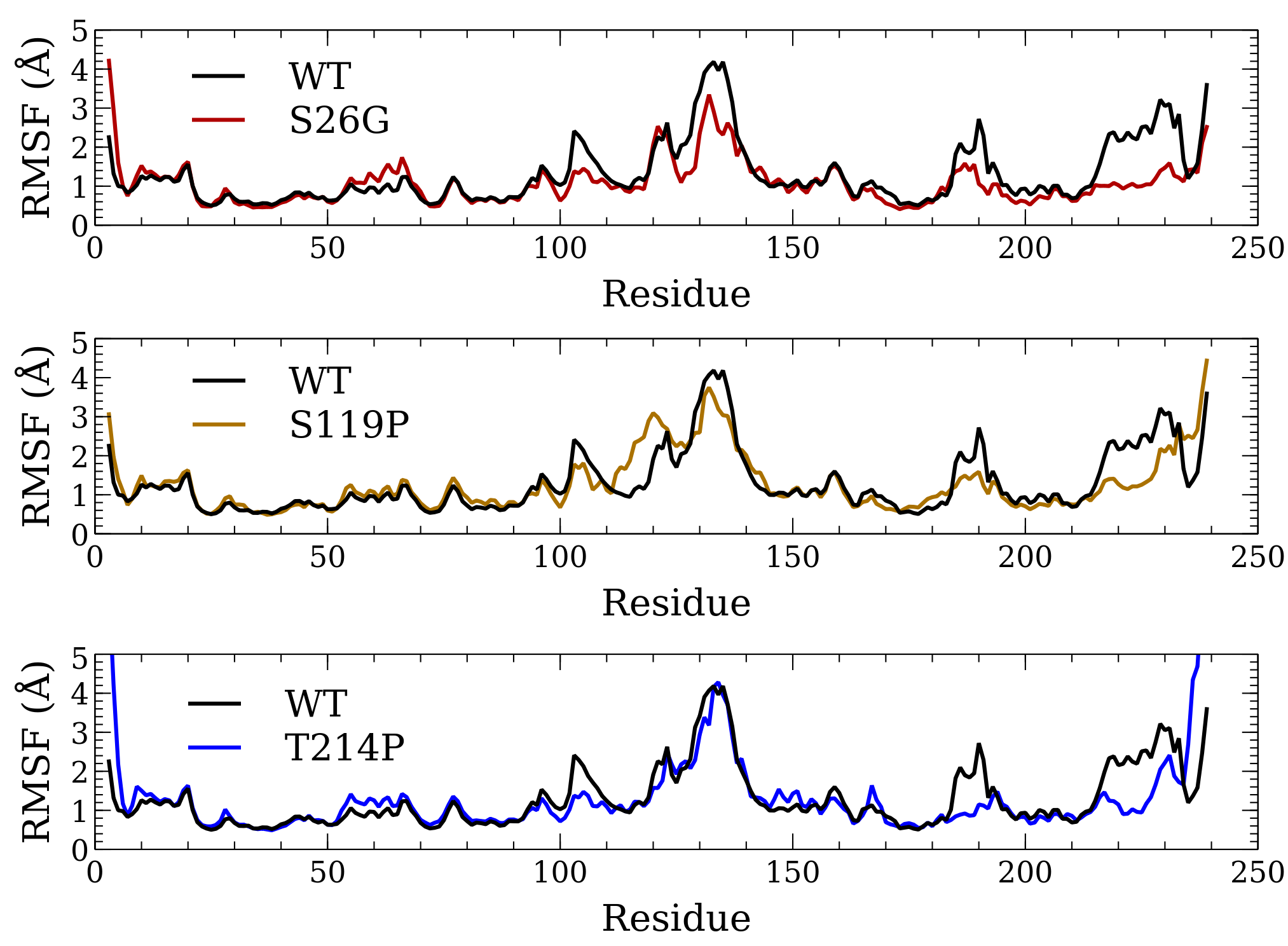

Supplement: Figure S6 — Root mean square fluctuation (RMSF) profiles for each mutant analyzed (S26G; S119P; T214P) compared to the GSTO-1 wild-type. [file Image_6.tiff]
